# From Waste to Healing Biopolymers: Biomedical Applications of Bio-Collagenic Materials Extracted from Industrial Leather Residues in Wound Healing

**DOI:** 10.3390/ma6051599

**Published:** 2013-04-29

**Authors:** Mercedes Catalina, Jaume Cot, Miquel Borras, Joaquín de Lapuente, Javier González, Alina M. Balu, Rafael Luque

**Affiliations:** 1 Institute of Advanced Chemistry of Catalonia, IQAC, CSIC, C/Jordi Girona 18-26, Barcelona 08034, Spain; E-Mails: jaume.cot@iqac.csic.es (J.C.); mborras@pcb.ub.cat (M.B.); jlapuente@pcb.ub.cat (J.L.); jgonzalezl@pcb.ub.cat (J.G.); 2 Department of Organic Chemistry, University of Córdoba, Campus de Rabanales, Edif. Marie Curie, Ctra. Nnal IV-A, Km 396, Córdoba E14014, Spain; E-Mails: z82babaa@uco.es (A.M.B); q62alsor@uco.es (R.L.)

**Keywords:** bio-collagen, leather waste valorisation, wound healing, tissue regeneration

## Abstract

The biomedical properties of a porous bio-collagenic polymer extracted from leather industrial waste residues have been investigated in wound healing and tissue regeneration in induced wounds in rats. Application of the pure undiluted bio-collagen to induced wounds in rats dramatically improved its healing after 7 days in terms of collagen production and wound filling as well as in the migration and differentiation of keratinocytes. The formulation tested was found to be three times more effective than the commercial reference product Catrix^®^ (Heal Progress (HP): 8 ± 1.55 *vs.* 2.33 ± 0.52, *p* < 0.001; Formation of Collagen (FC): 7.5 ± 1.05 *vs.* 2.17 ± 0.75, *p* < 0.001; Regeneration of Epidermis (RE): 13.33 ± 5.11 *vs.* 5 ± 5.48, *p* < 0.05).

## 1. Introduction

Wound healing is a complex process involving different events, cell types and signals. The initial stages comprise the formation of a blood clot and acute inflammatory response (1–3 days after wound infliction), followed by chronic inflammation (lymphocytes), proliferation and migration of dermal and epidermal cells, and collagen synthesis (days 4–7, scab formation; days 8–12, scab detachment and formation of new epidermis that becomes differentiated by day 12) [1,2]. Finally, tissue remodelling and differentiation occurs, leading to full recovery of the skin tissue (12–30 days) [1,2]. This reparation process is modulated by the interaction of molecular signals, primarily cytokines, which elicit and coordinate the different cellular activities which contribute to inflammation and healing [1,2,3]. Re-epithelialization is an essential step in the restoration of the epidermal barrier. This process can be considered as the result of three keratinocyte functions: migration, proliferation, and differentiation, being migration the most limiting [4]. A complex network of signaling factors and surface proteins need to be expressed and coordinated in the proper sequence in order to achieve keratinocyte motility and differentiation. This includes integrins, keratins, growth factors, cytokines and chemokines, eicosanoids, metals (Zn), oxygen tension, antimicrobial peptides, and matrix metalloproteinases, among others [5,6,7,8]. Keratinocyte migration and proliferation also depends on the interaction of keratinocytes with dermal fibroblasts and the extracellular matrix [7,8,9,10,11]. 

Advances in the knowledge of the aforementioned cell biology of wounds have prompted the search for new treatments that could improve or accelerate such healing process. 

In this sense, collagen was found to play a major role in wound healing [12,13]. The presence of collagen attracts fibroblasts (encouraging production of connective tissue), activates macrophages (stimulating formation of blood vessels), stimulates the growth of fibroblasts and keratinocytes and increases scar strength. These properties from collagen have been put to profit in collagen-based wound-dressing materials in order to improve and accelerate the healing process. In some cases, collagen natural sources (as in the case of the popular formulation Catrix^®^) have been utilized as compared to artificial dermal substitutes including Hyalomatrix^®^ [14]. Catrix^®^ is a collagen wound-healing powder, obtained from bovine tracheal cartilage, which has been shown to be effective in the treatment of wounds of different origins. It promotes the growth of fibroblasts and keratinocytes in the wound, prevents loss of fluid from the wound and protects it from bacterial infections and other agents. Catrix^®^ is biodegradable and therefore does not require removal from the wound bed before re-application [15].

We recently developed a novel and straightforward methodology to extract bio-collagen from a variety of residues from the leather waste industry [16]. Tailored-made biopolymers made of collagen could be isolated with high purity and formed into various interesting shapes including fibers, films and sponges which we hypothesized could have promising biomedical applications. 

In this preliminary work, a novel bio-collagenic derived biopolymer (hereafter denoted as COCAT) based on controlled hydrolytic degradation of the bio-collagen extracted from leather waste (e.g., bovine skin) has been tested for efficacy and safety in an induced wound-healing model in rats and its performance compared to that of a commercially available product (Catrix^®^). 

## 2. Results and Discussion

Collagenic biopolymers were isolated from pickled hides or skins and separated upon fundamental tanning operations including curing, liming, unhairing, deliming, bating and finally pickling (as well as the mechanical fleshing process). In this way, substances such as globular (globulin) albumin proteins, elastin, keratin, glycosaminoglycans (GAGs), hyaluronic acid, *etc.*, were quickly removed from the hides/skins leaving a final percentage of collagen content of over *ca.* 98% (dry weight). Subsequent treatment (detailed in the experimental section) [16] leaves a 3–5 wt % collagen (based on dry matter) from the original residues that was further purified by ultra filtration techniques and SDS-PAGE (***PolyAcrylamide*** Gel Electrophoresis). These showed the molecular weight range was placed over 200,000 Daltons, confirming a quite acceptable new regenerated collagen (COCAT).

The effectiveness of COCAT at different concentrations (5%, 10% and pure) in the wound-healing rat model was assessed in a comparative study with dry (no dressing) and wet (Carbopol^®^) control experiments as well as with a well-known commercial product for wound care (Catrix^®^). Catrix^®^ was approved by the FDA in 1998 and commonly used ever since, being a reference for wound care in different contexts. The results of several studies, as well as clinical experience, indicate that Catrix^®^ could be an optimal agent to stimulate the healing of chronic wounds because it exerts multiple actions in different phases of the healing process [17]. For these reasons, we choose Catrix^®^ as a reference substance to comparatively assess the performance of the biomedical application of COCAT in the wound-healing model.

**Figure 1:**
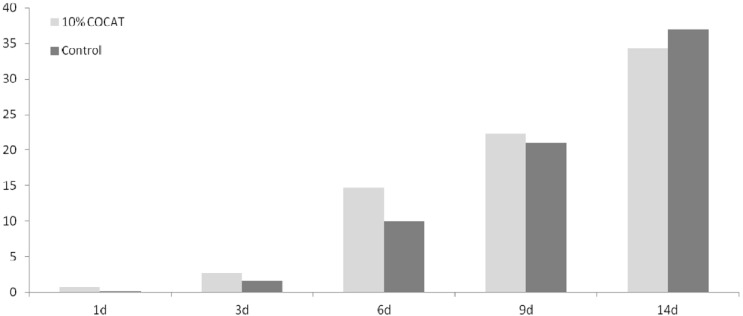
Mean Histological Score (MHS) at 1, 3, 6, 9 and 14 days after treatment.

It should be considered that this is a preliminary study, in which only a limited number of animals have been tested, and only a few, simple histological parameters assessed (see experimental details). The in-depth study of mechanisms is out of the scope of the present work. In the time-course study, throughout the test period the progress of healing was slightly higher in the COCAT-treated wounds than in controls, although the differences are not very important quantitatively (Figure 1, Table 1).

**Table 1:** Comparative and dose-response study, detail of histological observations.

Treatment	Mean Score
Control	12.2
Carbopol	7.8
Catrix	9.5
5% COCAT	15.0
10% COCAT	13.8
100% COCAT	28.8

However, a remarkable difference in wound healing was observed after 6 days, while at 9 days both sides (treated and control) were beginning to converge substantially towards complete healing. At 14 days, the values are equivalent. The histological observation showed that COCAT could indeed accelerate the healing process when applied at a concentration of 10%, presenting a difference of 5 points in the histological mean score after a 6 days treatment. At this time, re-epithelisation is at least incipient in treated animals, while has not perceptibly started in controls. Surprisingly, the macroscopic wound observation suggested that undressed wounds exhibited a better contraction and a faster reduction of its area. 

This discrepancy is not so uncommon: lack of agreement between macroscopical and histological assessment of wound healing has been reported by other authors [18]. These authors reported a study on burn healing in a porcine model, based on the assessment of re-epithelisation; although there was a good inter-observer agreement for both gross visual assessments (0.75) and histological observation (0.96), there was a poor agreement between gross visual and histological assessments of burn re-epithelialisation (−0.25). 

In our case, to explain this differences between macroscopic and histological observations we hypothesized that humidity could be responsible for this effect; this is the reason for the use of a “wet control” (Carbopol^®^ dressed wounds) in the comparative experiment. Carbopol^®^ are polymers of acrylic acid cross-linked with polyalkenyl ethers or divinyl glycol. These polymers are biologically inert, and are highly hydrophilic substances, not soluble in water. These properties make them suitable to maintain the wound humidified without introducing any biological activity. However, no significant differences were observed between dry control and Carbopol^®^-treated wounds.

**Figure 2:**
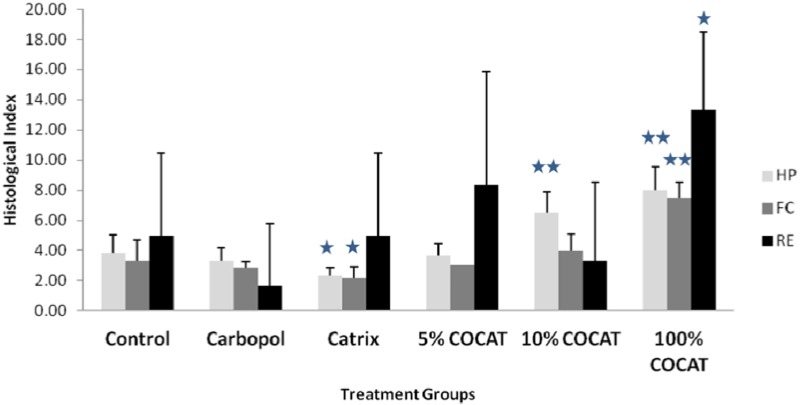
Histological Score at 7 days after treatment. HP: healing progress; FC: formation of collagen; RE: regeneration of epidermis. Statistical comparisons are made in all cases against the control. *: *p* < 0.05; **: *p* < 0.001.

**Figure 3:**
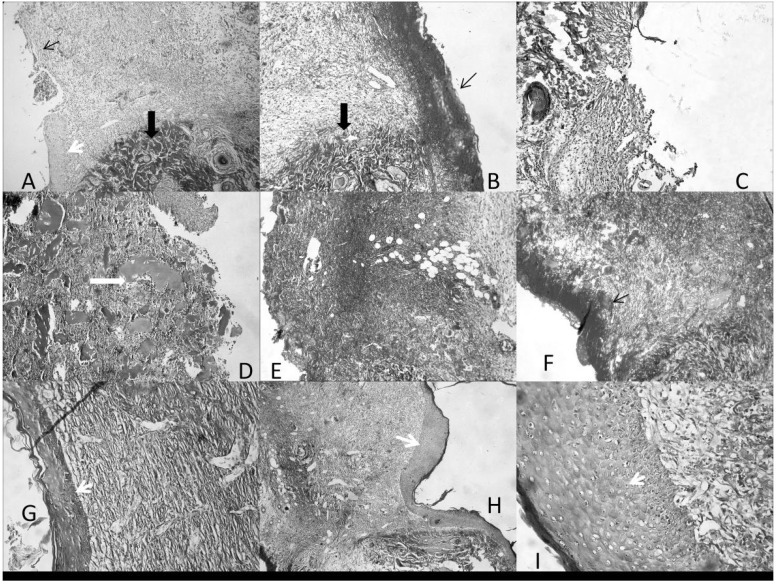
Histological images of the wound zone, with neighboring normal tissue. Cut at 5 µm in Paraplast, stained with AZAN Trichrome. 200 X. (**A**,**B**) Control; (**C**) Carbopol^®^; (**D**–**F**) Catrix^®^; (**G**–**I**) 100% COCAT. Thin arrow: presence (**B**,**F**) or absence (**A**) of scab; Thick black arrow: normal tissue; thick white arrow: collagen pools; White arrowhead: newly-formed epithelium.

The investigations were subsequently extended (Comparative and dose-response study) to various concentrations of COCAT, namely 5% and 100% (pure collagen), and compared again with the dry and wet control as well as with Catrix^®^. Results depicted in Figure 2 and Figure 3 showed that the pure 100% gelatinous form of COCAT provided a significantly advanced healing after 7 days treatment, with a histological mean score twice as high as that of the control experiments. The impact was remarkable on the three parameters considered for this study, but particularly related to re-epithelisation. As expected in any animal model, the variability of the response was relatively high but re-epithelization was even unexpectedly complete in some of the animals (Figure 3G–H).

## 3. Experimental Section

Bovine hides were supplied by the Leather Technology School of Igualada. Acetic acid (99.5% PS) and ammonia (25% PA) were supplied by Panreac. The basis for the preparation of the bioactive collagenic product (COCAT) was the degradation via hydrolysis of the extracted bio-collagen from leather waste under previous optimized conditions [16]. Briefly, grinded bovine hides in sizes as small as possible (First into squares of 2 cm × 2 cm and then into short fibers of 0.25 cm long) in a concentration of 50 g hide per liter of acid acetid (0.5 M) solution, were mixed by mechanical stirring (Heidolph stirrer) in a temperature controlled bath (Lauda E100) at 15 °C for 16 h. The grinding up offers significant save of chemicals, time and raised the yield of the reconstituted collagen.

The mild hydrolytic acid treatment is also important in the preparation of the biocollagenic polymer in yields of nearly 100%. Ultrafiltration (using a membrane of 10 KDa) was utilized in order to in order to remove salts and acetic acid residues and purify the collagenic biopolymers and SDS-PAGE (***PolyAcrylamide*** Gel Electrophoresis) to find out the molecular weight range. This was found to be over *ca.* 200 KDa , indicating a promising new regenerated collagen, the basis of the COCAT formulation. The COCAT product was then dried by lyophilization, using a freeze drier supplied by Telstar. Samples were frozen in an acetone/dry ice solution prior to the lyophilization.

Samples of different concentrations (5%, 10%) were prepared by mixing the lyophilized COCAT product in deionized water. In order to prepare the 100% COCAT product, an aliquot of the extracted bio-collagen (10 mL) was directly placed in a small Petri dish and allowed to air dry at a constant temperature (20 °C) and relative humidity (60%).

*In vivo* procedures were approved by the Institutional Animal Care and Use Committee (IACUC) of the Barcelona Science Park and by the IACUC of the Generalitat de Catalunya.

Female Wistar rats were used, of approximately 200 g (10–12 weeks of age) at the start of the experimental phase, in which a wound model was generated by performing controlled skin explants of 0.5 cm^2^ (0.5 cm × 1 cm), both at the back skin as well as at both sides of the spine. Each individual was performed two skin excision on both sides of the dorsal spine. To do this, with the animal under gaseous anesthesia (isoflurane; IsobaVet^®^), we proceeded to shave the dorsal region of the individual. After shaving, two rectangles of 0.5 cm^2^ (0.5 cm × 1 cm) were marked above the animal’s skin to define the perimeter of the resection to be made. With the help of a scalpel the flaps of skin (epidermis and dermis) were removed to reach the subcutaneous space while respecting the dorsal musculature. The surgical procedure was completed with disinfection of the wound generated and transfer of the animal to its cage for 24 h.

After 24 h of rest, the corresponding concentration of the products (no product-control-, COCAT and commercial ointment) was applied to the wounds of each animal under a dressing. The amount of product used was sufficient to completely cover the wound surface. After an hour of application, both products and control were removed and the animals were returned to their own routine. The health and welfare status of the animals through daily clinical observation (e.g., body weight, food intake, depositions and general behavior) was conducted and monitored on a daily basis. At the end of the assay, tissue samples corresponding to the wound site and surrounding area were fixed in 10% formalin and embedded in Paraplast.

Histological sections (5 μm) were obtained and then stained by the Azan trichrome for histopathological examination under a microscope Nikon Eclipse E400. To perform a semi-quantitative assessment of the histological results, the morphological parameters (HP: healing progress; FC: formation of collagen; RE: regeneration of epidermis) were rated on a scale of 0 to 10. The average was then obtained from all animals in each group. With regards to the epidermal regeneration, the number of individuals in each of three categories was recorded (S = yes, I = incipient, N = no), and values of 20, 10 and 0, respectively, were given, according to signs S, I and N. Finally, the average value of this parameter was added to the average values of progress of healing and collagen formation to obtain a Histological Mean Score (HMS). Observations were also made for inflammation and neovascularisation. Results were not recorded, as no relevant levels were observed in any case for these parameters.

Statistical analysis was performed by means of ANOVA (having previously assessed the normality of distribution and the homogeneity of variances with the Levenne Test), and group-to-group comparisons were made by the DMS Test. Results were compared for the HMS, but also for each one of the histological parameters recorded (HP, FC, RE) separately.

### 3.1. Experimental Design

A preliminary time-course study was performed on 15 animals, with only one concentration of the product (10%), to establish the time dynamics of wound healing in our experimental conditions and to determine the most suitable time point for the comparative study.

Afterwards, 6 test conditions were tested in a total of 18 animals, at day 7 after the treatment: control (no action, dry wound), Carbopol^®^ (control for wet conditions), Catrix^®^ (commercial wound dressing), test substance 5% solution, test substance 10% solution and test substance 100% (bio-collagen) (Table 2).

**Table 2:** Experimental Design, preliminary and main studies.

n	day	n of wound	Treatment
Time-course study			
15	1	3	Control
		3	10%
	3	3	Control
		3	10%
	6	3	Control
		3	10%
	9	3	Control
		3	10%
	14	3	Control
		3	10%
**Comparative and dose-response study**			
18	7	6	Control
		6	Carbopol^®^
		6	Catrix
		6	5% COCAT
		6	10% COCAT
		6	100 COCAT

## 4. Conclusions

In summary, results show that a bio-collagenic polymer extracted from leather industrial waste residues can have very interesting biomedical properties in tissue regeneration and wound-healing processes. Application of the pure undiluted bio-collagen to induced wounds in rats dramatically improved its healing after 7 days in terms of collagen production and wound filling as well as in the migration and differentiation of keratinocytes. This formulation was found to be three times more effective than the commercial reference product Catrix^®^.

Further experiments are currently ongoing in our laboratories to test the efficiency of COCAT in related biomedical applications but their translation of clinical tests and experimental trials in humans has been subjected to approval and will also follow in due course.
